# Acceptability of Pre-Exposure Prophylaxis and Non-Occupational Post-Exposure Prophylaxis among Men Who Have Sex with Men in Guilin, China

**DOI:** 10.3390/ijerph19063579

**Published:** 2022-03-17

**Authors:** Lingmi Zhou, Sawitri Assanangkornchai, Zhaohui Shi, Fusheng Jiang, Dong Yang, Wuxiang Shi

**Affiliations:** 1 Epidemiology Unit, Faculty of Medicine, Prince of Songkla University, Hat Yai, Songkhla 90110, Thailand; 6210330004@email.psu.ac.th; 2 Department of AIDS Control and Prevention, Guilin Center for Disease Control and Prevention, Guilin 541000, China; glcdcszh@163.com (Z.S.); glcdcjfs@163.com (F.J.); glcdcyd@163.com (D.Y.); 3 Health Management Unit, Faculty of Humanities and Management, Guilin Medical University, Guilin 541004, China

**Keywords:** HIV prevention, pre-exposure prophylaxis, non-occupational post-exposure prophylaxis, men who have sex with men, acceptability

## Abstract

Pre-exposure prophylaxis (PrEP) and non-occupational post-exposure prophylaxis (nPEP) are both effective strategies for preventing HIV. There is limited information about the acceptability of these prevention measures in undeveloped areas of China. We aimed to examine the acceptability of PrEP and nPEP and their determinants among men who have sex with men (MSM). 219 MSM were recruited in Guilin, China. In total, 28.6% (95% CI: 20.0–41.0) and 35.9% (95% CI: 27.3–49.5) of the participants had heard of PrEP and nPEP, respectively, while 57.0% (95% CI: 43.1–68.2) and 58.6 (95% CI:44.8–68.8) reported they would be willing to use PrEP and nPEP after the methods were explained. A higher acceptability of PrEP was seen among participants who were previously married (aOR = 3.30; 95% CI: 1.22–9.19), working as a laborer (aOR = 5.13; 95% CI: 1.64–17.59), migrant workers/farmers (aOR = 2.56; 95% CI: 1.15–5.79), government employees (aOR = 4.76; 95%CI: 1.80–13.02), had higher social support (aOR = 1.05; 95% CI: 1.03–1.08), and had been previously tested for HIV (aOR = 2.79; 95% CI: 1.36–5.94). A higher acceptability of nPEP was associated with those having higher social support (aOR = 1.06; 95% CI: 1.04–1.09), not knowing their sexual partner’s HIV status (aOR = 2.72; 95% CI: 1.23–6.12), and having a prior HIV test (aOR = 5.53; 95% CI: 2.58–12.51). PrEP and nPEP are acceptable, especially among MSM with higher social support and had received a previous HIV test. Effective education and different dissemination strategies to promote the acceptance of PrEP and nPEP among MSM are needed.

## 1. Introduction

Pre-exposure prophylaxis (PrEP) is the use of an antiretroviral drug on a regular basis to prevent the acquisition of human immunodeficiency virus (HIV) infection before exposure among uninfected people [1]. Non-occupational post-exposure prophylaxis (nPEP) refers to antiretroviral medicines taken after being potentially exposed to HIV occurring in a non-occupational manner, such as having sex without condoms and sharing needles with injecting drug users [2,3]. Since there is no HIV vaccine, PrEP and nPEP, as new and effective HIV infection-prevention measures, are worthy of being recommended to high-risk populations. A simulation study of HIV epidemics showed that, if 25% of the high-risk MSM in New York City used PrEP for five years, the incidence of new HIV infections could be reduced by 4% to 23% among the New York MSM population [4]. In the Asia–Pacific region, Thailand found that PrEP was cost-effective when it was administered to higher-risk MSM [5]. PrEP and nPEP are still new in China. In 2020, the first version of expert consensus on PrEP for HIV and Technical Guidelines for Post-Exposure Prevention of HIV (Trial) in China were published [6] and indicated that more studies are needed to better understand how both measures can be carried out in combination with other HIV-prevention strategies grounded on the local context. 

The severity of the HIV epidemic among MSM makes it an important public health issue. Although PrEP and nPEP may protect people at risk of HIV infection, the potential impact will only be realized if they are sufficiently acceptable to the at-risk populations so that they are used correctly and consistently. Most of the studies conducted in China were in big cities, such as Beijing, Wuhan, and Changsha [7,8,9,10,11,12], and few studies have focused on undeveloped areas. As PrEP and nPEP have been proven to be effective in preventing HIV transmission, a study conducted in Guilin, which is a third-tier city in the Guangxi autonomous region, would provide evidence to ensure equitable access to those HIV-prevention services. Early studies demonstrated that social support was highly associated with health service utilization, and a supportive social network was one of the facilitators of PrEP use among young adults [13,14]. However, few studies have investigated the behavioral and psychosocial correlations of acceptability of PrEP. Research on the behavioral and psychosocial correlations of nPEP acceptability is also lacking. In this study, we explored the psychosocial correlations of PrEP and nPEP acceptability. 

Guilin is located in the northeast of Guangxi, which ranks among the three most HIV-influenced provinces and suffers a disproportionate burden of HIV. At the end of 2020, there were reportedly more than 10,000 HIV-positive cases. In 2011, sentinel surveillance conducted in Guilin showed that the positive rate of HIV among MSM was 2.45%, which was higher than that in the general population (0.1%) [15]. The main HIV prevention methods in Guilin include behavioral intervention, while anti-retroviral treatment (ART) is provided to HIV-positive patients. However, these strategies may not be enough to curb the HIV/AIDS epidemic. According to results of MSM surveillance conducted from 2012 to 2016, the proportion of MSM who never used a condom when having heterosexual or homosexual sex showed an upward trend. Furthermore, the HIV infection rate increased from 3.0% in 2012 to 9.0% in 2016 [16]. Effective complementary prevention methods are thus needed. 

We undertook this exploratory study that aimed at examining the acceptability of PrEP and nPEP, and characterizing the influencing factors of the acceptability of PrEP and nPEP in a diverse sample of MSM in Guilin. We believe that our results can provide more evidence for health providers and policymakers for the further development of prevention strategies. 

## 2. Materials and Methods

### 2.1. Study Design and Setting

A cross-sectional survey among MSM was conducted in Guilin, China. We used a respondent-driven sampling (RDS) technique to enroll MSM from November 2020 to April 2021. 

### 2.2. Participants and Recruitment

The inclusion criteria for the participants were: male, aged 18 years or above, could give informed consent, lived in Guilin for at least one year, reported HIV-negative or status unknown, and had had oral or anal sex with men within the last 12 months before the interview. 

To recruit the participants, we provided a recruitment center in Guilin Center for Disease Control and Prevention (CDC), where staff had good rapport with MSM. One office in Guilin CDC was designated as the RDS room, which had the infrastructure for interviews and record-keeping. A quiet space for the interview was arranged to ensure privacy and confidentiality. At first, 55 MSM were recruited as the “seeds” of the RDS chain. Each seed was given three uniquely coded coupons, valid for two weeks, to recruit eligible participants from their social network. The new participants, after finishing the questionnaire, were given three coupons to recruit three new participants, and so on. Recruitment ended when the desired sample size was achieved. Each participant was compensated with 50 yuan (~8 US dollars) for their own participation in the study and 20 yuan (~3 US dollars) for the successful recruitment of each peer or partner into the study. To ensure the anonymity of the participants, eliminating the risk that signatures could be linked to responses, verbal informed consent was obtained before the interview in a voluntary and confidential manner. All information was kept in a locked cabinet. Electronic data were stored on a password-protected computer accessible only to the researchers. 

### 2.3. Variables and Measures

A structured interview questionnaire was used to elicit information regarding demographics, social–economic status, psychosocial factors, sexual behaviors, HIV literacy, current utilization of prevention services, and awareness and acceptability of PrEP and nPEP. 

Considering that PrEP and nPEP are still new to MSM in Guilin, a brief introduction about PrEP and nPEP was given, then awareness of PrEP and nPEP was assessed by the single question: “have you ever heard of PrEP/nPEP before this survey?”. Based on previous studies that assessed acceptability [17,18,19,20], we used “likely to use or likely to try a product” to reflect the likelihood of PrEP and nPEP acceptability, which was evaluated using a 7-point Likert scale. All participants, regardless of their awareness of PrEP and nPEP, were asked: “Overall, how likely would you use PrEP/nPEP?”, with possible responses ranging from 1 “very strongly unlikely” to 7 “very strongly likely”. The result was divided into three acceptability levels: 1–3 (low level), 3.1–5 (middle level), and 5.1–7 (high level) [21]. We defined those whose scores fell into the middle to high levels as accepting PrEP and nPEP. 

Demographic variables included age (at the time of survey), marital status, education, occupation, ethnicity, residence (urban or rural), household register, duration lived in Guilin, type of medical insurance (None, Urban employee basic medical insurance (UEBMI), Urban and rural resident basic medical insurance (URRBMI), and other), and tobacco and alcohol consumption (never smoke/drink, smoke/drink in the past, and current smoker/drinker).

Social–economic characteristics included the number of family members, personal monthly income, household monthly income, and household asset index. The household asset index was generated from self-reported household assets using principal component analyses, including ownership of a house/apartment, motor vehicle (car, truck, van, and SUV), motorbike, television, refrigerator, washing machine, personal computer, and smartphone. 

Psychosocial factors included perceived social support, perceived risk of HIV, knowing someone who has HIV, and homosexuality stigma. Social support was measured by the Multidimensional Scale of Perceived Social Support (MSPSS), Chinese version [22,23]. Emotional and instrumental support from family, friends, and significant others were assessed by a Likert scale, ranging from 1 (very strongly disagree) to 7 (very strongly agree). The results of all items were summed into a total score ranging from 12 to 84, with higher scores indicating higher perceived social support [24]. Homosexuality stigma was measured by the Chinese version of homosexuality-related stigma scales [25]. We assessed two domains in this study: public homosexual stigma (10 items) and self-homosexual stigma (8 items) using a 4-point Likert scale (1 = strongly disagree and 4 = strongly agree). The total score of all items ranged from 18 to 72, with higher scores indicating higher stigma. Cronbach’s alpha was 0.92 for the perceived social support and 0.84 for homosexuality stigma in the present study. HIV literacy was tested by a set of eight true/false questions, for instance, “HIV is a severe, fatal disease.” and “HIV cannot be cured.” If a participant provided at least six correct answers, he was considered to be HIV literate. 

Homosexual and heterosexual behavior, drug use, and the presence of sexually transmissible diseases were also assessed. Participants were asked to recall their homosexual and heterosexual behaviors in the past six months and condom use behaviors with each partner. We also assessed the total number and HIV status of the participants’ sexual partners in their lifetime. 

Current utilization of prevention services was measured by three yes/no items, including: (1) condom distribution or HIV counseling and testing, (2) community medication, maintenance treatment, and clean needle provision or exchange service, and (3) peer education in the last year. Any affirmative answer to the three items was considered as having used prevention services. 

HIV testing history and HIV status were asked during the interview. If a participant reported that he had been tested for HIV before, the result of HIV testing was assessed. 

### 2.4. Statistical Analysis

Data were entered using Epidata 3.1. We conducted descriptive analyses using RDSAT software (Respondent-Driven Sampling Analysis Tool; version 7.1.46) [26] to adjust potential bias in RDS due to the different social network size of respondents and homophily of recruitment [27]. The RDS-adjusted prevalence and 95% CI of the participant characteristics were calculated. We exported the sampling weights from RDSAT to conduct RDS-adjusted univariate and multivariate analyses. 

To determine factors associated with an ordinal degree of acceptability of PrEP and nPEP, ordinal logistic regression was used. Variables that had a Wald P-value less than 0.15 under univariate analysis were put into the initial multivariate logistic regression model, which was then finalized using a stepwise elimination approach. The results of each factor were presented in the form of an odds ratio and the corresponding 95% confidence interval (CI). In the final model, a variable with a Wald P-value less than 0.05 was declared as being statistically significant. All statistical analyses were performed in R version 4.1.0.

## 3. Results

### 3.1. Seed and Recruitment Chain Characteristics

The total sample included 219 MSM, including the 55 seeds. The majority of participants were recruited from three seeds, similar to prior RDS investigations [28]. One seed (M2) recruited the highest number of participants (*n* = 49), followed by M3 with 39, and M7 with 13 participants. These three chains were each five, six, and four waves long. Active seeds recruited 2.0 recruitment waves on average throughout the whole sample (range = 1–6).

### 3.2. Participant Characteristics 

The mean (SD) age was 35.6 (13.1) years. Most were single (68.1%) with senior high school and above education. Approximately 30% of the participants were employed in the business service. Most were of Han ethnicity and residing in urban areas. Over half had the Urban or Rural Residence Basic Medical Insurance Scheme. Most (62.7%, 95% CI: 53.8, 75.3) were non-smokers, while about half (47.6%, 95% CI: 33.4, 58.8) were current drinkers. The range of the household index was −1.38 to 1.02. All of the participants had equal to or above a 1500-yuan household income (Table 1).

### 3.3. Awareness and Acceptability of PrEP and nPEP

Of all participants, 28.6% and 35.9% reported having heard of PrEP and nPEP, respectively, as methods to prevent HIV and 15.2% and 2.0% had used PrEP and nPEP before. The mean scores of the acceptability scales of PrEP and nPEP were 4.06 (SD = 1.91) and 3.96 (SD = 1.86), respectively. Based on the cutoff point (score = 3.1), the overall acceptabilities of PrEP and nPEP were 57.0% (95% CI: 43.1, 68.2) and 58.6% (95% CI: 44.8, 68.8), respectively. Among those accepting PrEP and nPEP, 27.9% (95% CI: 19.6, 38.0) and 39.3% (95% CI: 30.0, 52.1) were in the middle level, and 28.8% (95% CI: 16.3, 37.9) and 19.1% (95% CI: 9.8, 25.8) were in the high level, respectively, (Table 2).

### 3.4. Social Support, Homosexual Stigma, Perceived Risk of HIV, and Knowing Someone Who Had HIV

The mean (SD) seven-item scales of social support and homosexual stigma were 56.4 (13.8) and 47.0 (7.7), respectively. More than half (65.1%) of the participants perceived their risk of HIV to be lower than the average. Most (83.9%) did not know anyone who had HIV. The mean (SD) number of MSM that the participants knew in Guilin was 23.3 (47.2). More than two-thirds (74.9%) knew five or fewer other MSM in Guilin (Table 3).

### 3.5. HIV-Related Knowledge and Behavior 

The overall HIV literacy rate was 72.9% (95% CI: 58.7, 84.7). In the past six months, the prevalence of having anal sex with men was 45.6% (95% CI: 34.0, 57.9). Among those who reported having anal sex with men, the majority (79–82%) used condoms during the last time they had sex, or used it whenever having anal sex. About half reported having anal sex with men 1–5 times in the previous week, while 9.0% (95% CI: 1.6, 26.4) had commercial sex with men. 

Of all participants, 15.8% (95% CI: 9.0, 22.7) had sexual intercourse with a female. Among these, 59.8% (95% CI: 49.3, 100.0) never used a condom when they had sex with a female. Most participants (88.4%; 95% CI: 81.2, 92.9) reported having five or fewer sexual partners in their lifetime and 3.0% (95% CI: 0.2, 6.6) had had an HIV-positive sexual partner. None of the participants had used drugs before. Less than 1% had been diagnosed with an STD in the last year (Table 4).

### 3.6. HIV Prevention Service Usage

Of all participants, 80.9% (95% CI: 80.5, 91.0) had used at least one of the prevention services in the past year, while 77.1% (95% CI: 68.6, 88.1) were tested for HIV before this survey. Among the HIV-tested participants, 94.0% (95% CI: 91.1, 99.1) reported themselves as HIV negative, while the rest did not know their test result (Table 4).

### 3.7. Factors Associated with Acceptability of PrEP 

In the univariate analysis, the type of occupation, medical insurance, household asset index, social support, and HIV testing were significantly associated with the acceptability of PrEP. Ten variables with a Wald P-value less than 0.15 under univariate analysis were included in the initial multivariate model. 

Based on the multivariate analysis, only four significant variables, namely marital status, type of occupation, social support, and HIV testing remained significant in the final model. Compared with single MSM, the odds of attaining a higher acceptability level among participants who were separated/divorced/widowed was 3.3 (95% CI:1.22, 9.19). Laborers, migrant workers/farmers, and government employees were 2–5 times as likely to be in the higher level of PrEP acceptability, compared to those in business services. Having one higher social support score increased the likelihood of accepting PrEP by about 5% (1.05, 95% CI: 1.03, 1.08). Lastly, those who had ever been tested for HIV were 2.79 times (95% CI: 1.36, 5.94) more likely to have a higher acceptability of PrEP (Table 5).

### 3.8. Factors Associated with Acceptability of nPEP

The univariate ordinal logistic regression analysis revealed eight variables significantly associated with the acceptability of nPEP, namely marital status, occupation type, duration lived in Guilin, type of medical insurance, alcohol-drinking status, social support score, HIV-positive sexual partner, and testing for HIV before. 

In the multivariate analysis, participants who were married or cohabiting were 64% less likely to have a higher acceptability of nPEP compared to those who were single (AOR = 0.36; 95% CI: 0.15, 0.81). Those who were unaware of their sexual partner’s HIV status were 2.72 times (95% CI: 1.23, 6.12) more likely to have higher acceptability compared to those whose partner was HIV negative, while those who had a prior history of testing for HIV were 5.53 times (95% CI: 2.58, 12.51) more likely. However, those who had HIV-positive sexual partners were significantly less likely to accept nPEP. Finally, the odds of increasing the acceptability by one or more level was 1.06 for each additional unit of social support (AOR = 1.06; 95% CI: 1.04, 1.09) (Table 6).

## 4. Discussion

This study aimed to evaluate the acceptability of PrEP and nPEP and associated factors in a diverse sample of MSM recruited using a respondent-driven sampling technique. In general, the MSM recruited in this study were middle-aged adults, single, and well-educated, and lived in urban areas of Guilin. They perceived that their risk of HIV was low. About one-third had heard of PrEP or nPEP, after being well informed, while more than half showed moderate to high interest in using PrEP or nPEP. The degrees of acceptability to both PrEP and nPEP were significantly determined by socio-economic factors, e.g., marital status, extent of social support, and history of a previous HIV test. 

MSM in Guilin showed low awareness of both PrEP (28.6%) and nPEP (35.9%). Compared to studies in other parts of China, the level of PrEP awareness was higher than the level estimated in Guangxi in 2017 (22.1%) [29], but both were much lower than the rates in developed areas of China (43.1% for PrEP) [30], Beijing (42.5% for nPEP) [12], and large cities of China (61% for nPEP) [10]. Compared to other low- and middle-income countries (29.7%) [31], our study showed similar awareness of PrEP among MSM. However, the awareness was unquestionably lower than that in countries where PrEP and nPEP are available, i.e., 43.9% and 96.0%% for PrEP in the US and Canada, and 82.3% and 80.2% for nPEP in Italy and New York City, respectively [32,33,34,35]. A plausible explanation for the difference might be the increasing number of exploratory studies and the promotion of PrEP and nPEP carried out in recent years, which have mainly focused on developed cities in China. Guilin is an undeveloped city, but suffers from a disproportionately higher burden of the HIV epidemic in China. There is also a lack of a structured effort to inform the population of the existence of both PrEP and nPEP [12].

Our study found that, once the participants were well informed about PrEP and nPEP, their acceptability for both techniques was high (57.0% and 58.6% for PrEP and nPEP, respectively) [36,37,38]. The level of acceptability for PrEP was consistent with that among MSM observed in a meta-analysis of 68 studies (57.8%) [39]. However, compared to another Chinese study conducted in four large cities (Beijing, Changsha, Guangzhou, and Shanghai) (70%) [10], the acceptability of nPEP among the MSM population in Guilin was lower. This implies that both PrEP and nPEP were fairly acceptable among MSM; however, more research to explore the acceptability of PrEP and nPEP barriers is needed for informing policymakers of the best ways to provide service delivery in the future.

Those who were previously married were more likely to accept PrEP; however, those who were currently married or cohabiting were less likely to accept nPEP compared to single MSM. This might be related to the different risk engagement behaviors between these groups. People without stable partners may be more likely to engage in risky behaviors and have a higher proportion of HIV infection [40], thus desiring to engage in this self-protective method. 

In our study, different occupations showed different levels of acceptance for PrEP. To our knowledge, most of the related studies did not report the association between occupation and the acceptability of PrEP [7,9,41]. This highlights an area for future research to clarify the role of occupation in influencing willingness to use PrEP. Our results also suggest that different acceptance promotion strategies should be piloted for different occupational groups. 

The extent of social support was a significant factor affecting the acceptability of PrEP and nPEP in this study, a result consistent with a study from Thailand [42]. However, other studies showed that some participants were afraid that their partner would refuse to accept their use of PrEP [43,44]. In our study, the mean score of social support was lower than that in other studies [45,46,47]. A good relationship with family, friends, and significant others may ease the context of stigma towards PrEP and nPEP. Another study indicated that friends’ supportive attitudes towards PEP increased the willingness to use PEP [10]. Therefore, when conducting prevention strategies, healthcare workers should consider the positive effect of social support and the leveraging power of partners and peers of MSM to facilitate access and utilization of PrEP and nPEP [31,48]. However, we did not elicit the source of “social support”, whether this was largely represented by friends, families, or other significant supportive peers. Further research to explore the sources of social support would be beneficial in promoting the acceptability of PrEP and nPEP [49].

MSM with partners of unknown HIV status were more likely to accept nPEP, which may partly be because nPEP is more attractive to people at high risk of HIV infection. The estimated HIV prevalence of MSM with partners of unknown HIV status was approximately 11% [50]. However, MSM who had HIV-positive partners showed less interest in nPEP. A possible explanation for this is that people living with HIV (PLWH) can easily obtain free antiretroviral therapy (ART) in China under the “treatment as prevention” strategy. PLWH who have suppressed viral loads have a low risk of HIV transmission [51], and MSM may, therefore, perceive that their risk of HIV infection is low despite living with them. However, the influence of partners’ HIV status on the acceptability of PrEP was not found in this study, indicating that promotion strategies for PrEP and nPEP may differ. When promoting nPEP, healthcare workers should emphasize the efficacy of risk behavior modification; however, to increase the use of PrEP, we suggest that healthcare workers be more concerned with the protective benefits of PrEP. 

The association between HIV testing and acceptability of PrEP and nPEP may be related to higher health awareness among MSM who had tested for HIV before. A previous study showed that HIV testing was associated with higher PEP awareness [34]. Those who had a prior HIV test may be more familiar with healthcare services. Furthermore, HIV testing clinics may be a first point for promoting education and awareness for PrEP and nPEP, as they may be a point that reaches a large group of HIV risk cases. This suggests that voluntary HIV counseling and testing services could be used to disseminate prevention information. 

There are certain limitations to our study. First, as this was a cross-sectional study, we could only infer the association of factors for the acceptability of PrEP and nPEP, but could not determine causality. Second, this study was conducted in one city; therefore, caution should be used when generalizing the findings to MSM populations in regions that are dissimilar to Guilin. Third, behavioral information depended on self-reporting, which may be affected by information bias, such as recall and social desirability bias. Furthermore, there has been no consensus regarding the operational definition of acceptability; therefore, we used the term “likely to use” to reflect acceptability based on some other studies. When conducting the survey, there was no PrEP program and formal usage specification of nPEP available in Guilin. We could only assess the hypothetical acceptability of these drugs. The gap between hypothetical and actual acceptability should be considered when interpreting our results. We also did not ask the respondents their willingness to pay for PrEP. Before the implementation of prevention strategies, further studies examining willingness to pay are needed. Lastly, we did not collect biospecimens to verify whether the participants were truly HIV-negative.

## 5. Conclusions

The acceptability of PrEP and nPEP may be affected by background characteristics, as well as social and behavioral factors, but neither individual risk factors, nor the perceived risk of HIV, were observed to be influential factors of acceptability of those prevention medicines. Our findings suggest that PrEP and nPEP were acceptable among MSM; however, the promotion of these preventions remains challenging. Studies based on the acceptability of PrEP and nPEP will be important to elaborate on the facilitators and barriers to implementing new prevention strategies. Effective education and various dissemination strategies to promote the acceptance of PrEP and nPEP among Chinese MSM are needed. 

## Figures and Tables

**Table 1:** Crude and adjusted characteristics among men who have sex with men in Guilin, China (*n* = 219).

	N (%)	Weighted% (95%CI)
Age		
≤25 years	60 (27.4)	44.3 (24.9, 56.6)
25~35 years	63 (28.8)	31.9 (21.7, 45.3)
>35 years	96 (43.8)	23.9 (14.8, 39.6)
Marital status		
Single	158 (72.1)	68.1 (55.9, 78.4)
Married or cohabited	40 (18.3)	20.9 (12.0, 31.7)
Separated/Divorced/Widowed	21 (9.6)	11.0 (5.2, 18.4)
Education		
Primary school and below	37 (16.9)	19.5 (11.8, 32.5)
Junior high school	17 (7.8)	13.2 (4.5, 23.5)
Senior high school and above	165 (75.3)	67.3 (51.3, 78.6)
Occupation		
Business person	70 (32.0)	29.9 (19.5, 39.6)
Laborer	23 (10.5)	7.4 (2.9, 17.4)
Migrant worker/farmer	27 (12.3)	18.2 (7.9, 27.6)
Government employee	21 (9.6)	8.5 (2.3, 17.0)
Retired/houseworker/unemployed	25(11.4)	6.5 (3.0, 10.6)
Other	53 (24.2)	29.6 (17.8, 43.3)
Ethnicity		
Han	191 (87.2)	83.5 (73.5, 93.6)
Other	28 (12.8)	16.5 (6.4, 26.5)
Urban resident	177 (80.8)	77.1 (66.9, 87.3)
Household register		
Guilin City	95 (43.4)	38.5 (24.2, 49.5)
Guilin County	71 (32.4)	31.0 (24.8, 46.8)
Other	53 (24.2)	30.5 (18.1, 38.4)
Duration lived in Guilin (years)		
1–20	128 (58.4)	67.6 (54.1, 78.1)
20–40	61 (27.9)	24.4 (14.6, 36.7)
>40	30 (13.7)	8.1 (2.3, 17.3)
Medical insurance		
None	17 (7.8)	13.1 (3.3, 22.0)
UEBMI ^a^	44 (20.1)	20.2 (9.8, 31.2)
URRBMI ^b^	141 (64.4)	59.9 (48.3, 74.1)
Other	17 (7.8)	6.8 (3.0, 13.7)
Number of family members		
1–3	114 (52.1)	46.9 (38.8, 63.3)
>3	105 (47.9)	53.1 (36.7, 61.2)
Personal income ≥1500 yuan per month	203 (92.7)	91.0 (82.9, 97.1)

^a^ UEBMI = Urban employee basic medical insurance. ^b^ URRBMI = Urban or rural residence basic medical insurance.

**Table 2:** Crude and adjusted awareness, use, and acceptability of PrEP and nPEP.

	PrEP		nPEP	
	N (%)	Adjusted% (95% CI)	N (%)	Adjusted% (95% CI)
Awareness	81 (37.0)	28.6 (20.0, 41.0)	124 (56.6)	35.9 (27.3, 49.5)
Use	3 (3.7)	15.2 (0.0, 37.9)	5 (4.0)	2.0 (0.0, 6.1)
Acceptability				
Low level	75 (34.2)	43.3 (32.9, 57.3)	80 (36.5)	41.6 (30.3, 54.6)
Medium level	84 (38.4)	27.9 (19.6, 38.0)	86 (39.3)	39.3 (30.0, 52.1)
High level	60 (27.4)	28.8 (16.3, 37.9)	53 (24.2)	19.1 (9.8, 25.8)

**Table 3:** Crude and adjusted awareness, psychosocial factors, and behavior among men who have sex with men in Guilin, China (*n* = 219).

	N (%)	Adjusted % (95% CI)
Social support (mean (SD))	56.37 (13.789)	
Perceived risk of HIV		
Lower than average	151 (68.9)	65.1 (52.2, 77.4)
Average	61 (27.9)	27.8 (16.4, 39.8)
Higher than average	7 (3.2)	7.1 (1.9, 14.5)
Knowing someone who had HIV		
Don’t know	155 (10.8)	83.9 (68.9, 89.8)
Yes, my family member	5 (2.3)	0.9 (0.1, 2.0)
Yes, my friend or acquaintance	59 (26.9)	15.2 (9.2, 30.1)
Number of MSM known in GL ^a,b^		
≤5	67 (30.6)	74.9 (64.2, 80.3)
6–10	64 (29.2)	17.1 (12.5, 24.7)
>10	87 (39.7)	8.0 (6.0, 12.7)
Homosexuality stigma (mean (SD))	47.00 (7.733)	

^a^ GL: Guilin; ^b^ one missing.

**Table 4:** Crude and adjusted HIV-related knowledge and behavior among men who have sex with men in Guilin, China (*n* = 219).

	N (%)	Adjusted % (95% CI)
HIV literacy		
0–5	40 (18.3)	27.1 (15.3, 41.3)
6–8	179 (81.7)	72.9 (58.7, 84.7)
Had anal sex with men in the last six months.	129 (58.9)	45.6 (34.0, 57.9)
Used condoms during the last time they had anal sex with men	105 (47.9)	82.1 (59.6, 92.2)
Had commercial sex with men in the last six months	9 (4.1)	9.0 (1.6, 26.4)
Had sexual intercourse with female in the past six months	42 (19.2)	15.8 (9.0, 22.7)
Number of sexual partners in lifetime ^a^		
≤5	169 (77.2)	88.4 (81.2, 92.9)
6–10	32 (14.6)	8.9 (4.4, 14.9)
>10	17 (7.8)	2.7 (0.8, 6.6)
Had HIV-positive sexual partner	9 (4.1)	3.0 (0.2, 6.6)
Diagnosed with an STD in the previous year	6 (2.7)	0.7 (0.1, 1.5)
Used HIV prevention service	179 (81.7)	80.9 (80.5, 91.0)
Condom promotion and distribution/HIV counseling and testing	169 (77.2)	73.6 (65.0, 85.0)
Community medication maintenance treatment/clean needle provision/exchange	2 (0.9)	0.0 (-)
Peer education	142 (64.8)	61.9 (54.7, 76)

^a^ one missing.

**Table 5:** Univariate and multivariate ordinal logistic regression analysis of factors associated with the acceptability of PrEP.

	Medium ^a^	High ^b^	P-Value ^c^	AOR (95% CI) ^d^	P-Value	Wald’s P-Value
Age (years)			0.101			
≤25	26.3 (10.8, 40.1)	24.6 (10.6, 39.6)		-		
25~35	31.0 (12.0, 43.8)	26.1 (5.0, 49.7)		-		
>35	29.4 (12.8, 48.7)	25.8 (8.8, 38.6)		-		
Marital status			0.120			0.010
Single	28.6 (19.6, 42)	30.3 (13.5, 43.4)		1.00		
Married or cohabiting	17.5 (6.9, 36.1)	22.7 (4.3, 43.4)		0.56 (0.26, 1.22)	0.150	
Separated/divorced/widowed	41.6 (12.9, 74.1)	32.4 (10.7, 59.9)		3.30 (1.22, 9.19)	0.021	
Occupation			0.001			0.010
Businessperson	32.2 (12.1, 43.1)	18.1 (8.3, 32.8)		1.00		
Laborer	14.7 (1.0, 39.2)	70.7 (12.0, 93.7)		5.13 (1.64, 17.59)	0.007	
Migrant worker/Farmer	28.3 (12.4, 72.9)	34.3 (6.4, 69.9)		2.56 (1.15, 5.79)	0.023	
Government employee	52.5 (11.6, 94.5)	33.5 (0.0, 62.1)		4.76 (1.80, 13.02)	0.002	
Residence in rural area	16.0 (4.0, 32.9)	22.8 (7.2, 36.9)	0.090	-		
Medical insurance			0.012			
None	31.0 (10.3, 86.1)	47.0 (6.3, 82.4)		-		
UEBMI ^e^	33.3 (8.6, 64.9)	32.7 (4.4, 62.5)		-		
URRBMI ^f^	30.0 (18.8, 39.8)	21.6 (12.1, 30.4)		-		
Other	19.0 (1.3, 45.7)	47.1 (16.0, 82.8)		-		
Personal income ≥1500 yuan per month	30.2 (18.9, 40.3)	30.6 (16.4, 41.6)	0.073	-		
Household asset index	_	_	0.009	-		
Social support	_	_	0.000	1.05 (1.03, 1.08)	0.000	0.000
Have HIV-positive sexual partner			0.149			0.103
No	27.3 (18.5, 40.3)	30.9 (15.5, 40)		1.00		
Yes	15.0 (0.0, 100)	9.0 (0.0, 79.5)		0.09 (0.01, 0.62)	0.035	
Unknown	27.6 (10.7, 52.6)	26.6 (13.2, 47.7)		0.90 (0.40, 1.98)	0.789	
Tested for HIV before	29.8 (19, 39.9)	32.1 (17.5, 42.7)	0.001	2.79 (1.36, 5.94)	0.007	0.007

^a^ Medium level of acceptability of PrEP, row percentages. ^b^ High level of acceptability of PrEP, row percentages. ^c^ Wald’s P-value for univariate ordinal logistic regression. ^d^ AOR = Adjusted odds ratio. 95% CI = confidence interval. ^e^ UEBMI = Urban employee basic medical insurance. ^f^ URRBMI = Urban or rural residence basic medical insurance.

**Table 6:** Univariate and multivariate ordinal logistic regression of factors associated with acceptability of nPEP.

	Medium ^a^	High ^b^	P Value ^c^	AOR (95% CI) ^d^	P Value	Wald P Value
Marital status			0.015			0.005
Single	42.9 (29.5, 55)	17.6 (7.0, 27.7)		1.00		
Married or cohabiting	25.5 (7.4, 50.3)	14.7 (2.0, 21.7)		0.36 (0.15, 0.81)	0.016	
Separated/divorced/widowed	47.1 (17.5, 77.4)	34.5 (11.9, 65.3)		2.28 (0.85, 6.14)	0.102	
Occupation			0.015			
Business service	39.5 (19.4, 50.6)	13.7 (6.9, 26.5)		-		
Laborer	10.9 (0.5, 30.4)	68.9 (13.4, 89.2)		-		
Government employee	57.7 (5.4, 88.6)	13.7 (0.0, 25.9)		-		
Household register			0.121			
Guilin city	35.1 (19.8, 56)	22.5 (5.2, 37.4)		-		
Guilin country	58.0 (36.1, 77.4)	10.7 (3.7, 19.8)		-		
Others	31.4 (18.4, 55.4)	16.0 (4.8, 26.9)		-		
Time lived in Guilin (Years)			0.002			0.102
1–20	38.4 (28.2, 53.8)	14.2 (7.2, 19.6)		1.00		
20–40	42.5 (22.4, 70)	27.6 (3.7, 41.6)		1.89 (0.99, 3.62)	0.056	
>40	46.0 (1.8, 85.4)	20.2 (0.0, 44.8)		1.99 (0.76, 5.25)	0.163	
Medical insurance			0.032			
None	62.8 (20.0, 96.7)	4.9 (0.6, 22.7)		-		
UEBMI ^e^	35.7 (8.9, 64.6)	27.8 (3.4, 60.0)		-		
URRBMI ^f^	44.4 (30.9, 58.7)	14.0 (6.6, 19.2)		-		
Others	16.6 (0.5, 38.4)	39.7 (11.3, 80.8)		-		
Alcohol consumption			0.012			0.054
Never drink	38.7 (20.3, 63.5)	13.1 (4.6, 21.0)		1.00		
Drink in the past	31.2 (13.3, 61.5)	41.2 (12, 51.2)		1.67 (0.72, 3.91)	0.234	
Current drinker	42.5 (26.4, 58.1)	17.3 (4.9, 32.4)		0.62 (0.33, 1.17)	0.142	
Household asset index	_	_	0.058	_		
Social support	_	_	0.000	1.06 (1.04, 1.09)	0.000	0.000
Have HIV-positive sexual partner			0.049			0.004
No	37.2 (24.6, 50.2)	19.6 (8.8, 27.6)		1.00		
Yes	46.3 (0.9, 100)	0		0.16 (0.02, 0.76)	0.032	
Unknown	44.4 (23.3, 73.9)	27.8 (12.4, 46.7)		2.72 (1.23, 6.12)	0.015	
Tested for HIV before	25.8 (12.3, 61.0)	0	0.000	5.53 (2.58, 12.51)	0.000	0.000

^a^ Medium level of acceptability of PrEP, row percentages. ^b^ High level of acceptability of PrEP, row percentages. ^c^ Wald’s P-value for univariate ordinal logistic regression. ^d^ AOR = Adjusted odds ratio. 95% CI = confidence interval. ^e^ UEBMI = Urban employee basic medical insurance. ^f^ URRBMI = Urban or rural residence basic medical insurance.
